# Finding the needle in the haystack: novel multi-omics approach readily identifies candidate resistance genes of Tartary buckwheat against Rhizoctonia solani infection

**DOI:** 10.1093/plcell/koad133

**Published:** 2023-05-13

**Authors:** Mariana Schuster

**Affiliations:** Assistant Features Editor, The Plant Cell, American Society of Plant Biologists, USA; Leibnitz Institute of Plant Biochemistry, Halle (Saale), 06120, Germany

Plant diseases caused by fungal pathogens challenge both natural ecosystems and agricultural settings, with the latter making fungal diseases a hazard to global food security. Currently, control measures for phytopathogenic fungi revolve around the application of chemical fungicides, though the overuse of such fungicides can encourage the generation of fungicide-insensitive strains that may threaten both plant and human health (Fisher et al. 2020). Thus, safe and durable resistance to fungal pathogens is needed to sustainably tackle the challenge of fungal diseases in plants. The creation of resistant crop varieties requires knowledge of the mechanisms of fungal resistance in the host plants. Therefore, identifying resistance genes is a key step toward the sustainable management of fungal diseases. In this issue, Yuqi He and colleagues (He et al. 2023) report an innovative and efficient strategy for the identification of novel resistance genes in Tartary buckwheat (*Fagopyrum tataricum*). The authors used a multi-omics approach to fish for resistance genes from buckwheat against the devastating and complex fungal pathogen *Rhizoctonia solani*. They found 2 candidate resistance genes and provided data sets that should aid the identification of many more.

**Figure 1.**
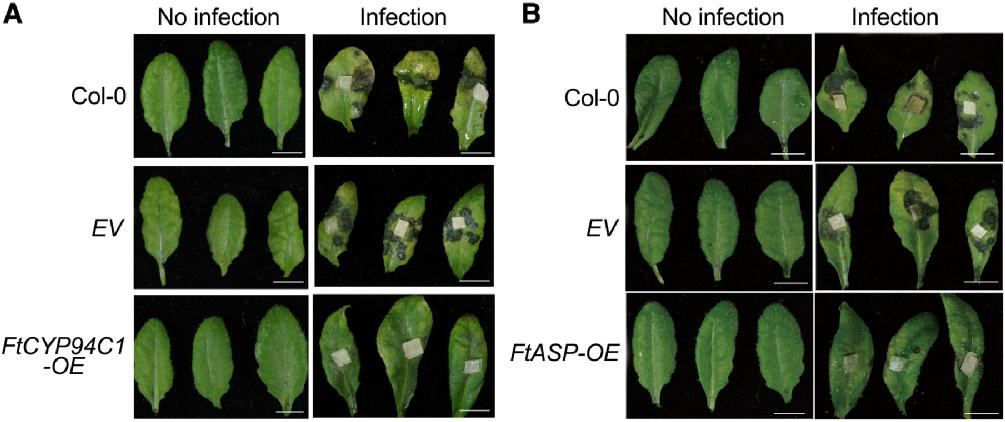
Heterologous expression of candidate resistance genes of Tartary buckwheat in Arabidopsis confers resistance to *R. solani*. Phenotype of Arabidopsis expressing either *FtCYP94C1* (**A**) or *FtASP* (**B**) infected with *R. solani* isolate AG4-HGI 3. Detached leaves of 2-week-old Arabidopsis seedlings were inoculated with subcultured mycelial disks for 2 days. Arabidopsis leaves of wild-type (Col-0) or from lines transformed with the empty vector control (EV) were used as negative controls. The phenotypes were observed in leaves of 3 Arabidopsis seedlings (*n* = 3). The experiments were performed 3 times using different batches of Arabidopsis seedlings. Photographs from 1 representative experiment are shown. Scale bars, 1 cm. Adapted from He et al. (2023), Figures 4 and 6.

The wide host range *R. solani* isolate AG4-HGI 3 was obtained from Tartary buckwheat, in which the pathogen causes severe yield loss due to disease (Li et al. 2021). To gain insights into its pathogenicity and potential buckwheat resistance genes, the authors performed whole genome sequencing and transcriptomic analysis of this isolate on Tartary buckwheat. The resulting genome sequence and fungal transcriptomics data constitute a rich resource of candidate virulence determinants for this pathogen. The authors analyzed the plant genes in this data set to identify candidate resistance genes of Tartary buckwheat to AG4-HGI 3. Enrichment analysis highlighted the role of hormonal pathways during defense. To test this hypothesis, the authors evaluated the disease resistance of Tartary buckwheat seedlings pretreated with different plant hormones and discovered that jasmonic acid treatment increased resistance to AG4-HGI 3. Comparative analysis of time-dependent transcriptome changes in Tartary buckwheat following methyl jasmonate treatment identified a jasmonic acid signaling transduction gene, cytochrome P450 *FtCYP94C1*, as a putative resistance gene. Overexpression of this gene in Tartary buckwheat and Arabidopsis (*Arabidopsis thaliana*) resulted in enhanced disease resistance in both cases (Figure 1A). To better understand the mechanism of disease resistance mediated by *FtCYP94C1*, the authors analyzed the metabolome of *FtCYP94C1* Arabidopsis overexpression lines and discovered that increased resistance is likely due to increased accumulation of disease resistance–related flavonoids.

To further mine for resistance genes in buckwheat, He et al. combined their transcriptomic analysis with a genome-wide association study by investigating the disease index of 360 Tartary buckwheat accessions in their interaction with *R. solani*. This resulted in the identification of 49 more candidate resistance genes, including the aspartic proteinase *FtASP*, which increased Arabidopsis disease resistance when overexpressed (Figure 1B). The authors determined that enhanced disease resistance caused by *FtASP* is due to its effect of directly inhibiting the growth of *R. solani* and that this function is not related to its protease activity (because the FtASP was inactive as a protease) but is instead caused by an antimicrobial peptide in the N-terminus of the protein.

This study offers a robust and holistic strategy for identifying virulence determinants in a genetically complex system such as *R. solani*. Moreover, the work highlights the power of combining genome-wide association study and transcriptomics to find resistance genes in plants and provides a valuable list of both candidate virulence determinants and candidate resistance genes for the economically relevant Tartary buckwheat–*R. solani* pathosystem.
